# 10th Annual Symposium on Self-Monitoring of Blood Glucose, April 27–29, 2017, Warsaw, Poland

**DOI:** 10.1089/dia.2017.0356

**Published:** 2018-01-01

**Authors:** Christopher G. Parkin, Anita Homberg, Rolf Hinzmann

**Affiliations:** ^1^ CGParkin Communications, Inc., Boulder City, Nevada.; ^2^ Roche Diabetes Care GmbH, Mannheim, Germany.

**Keywords:** Continuous Glucose Monitoring, Self-Monitoring of Blood Glucose, mHealth, Closed-loop

## Abstract

International experts in the field of diabetes and diabetes technology met in Warsaw, Poland, for the 10th Annual Symposium on Self-Monitoring of Blood Glucose. The goal of these meetings is to establish a global network of experts to facilitate new collaborations and research projects that can improve the lives of people with diabetes. The 2017 meeting comprised a comprehensive scientific program, parallel interactive workshops, and four keynote lectures.

## Opening Lecture

### News from the World of Diabetes

Satish Garg, University of Colorado Denver, Aurora, Colorado

#### Cardiovascular outcome trials—update

Numerous studies have shown that intensification of insulin treatment confers significant glycemic benefits. However, the effects of this treatment approach on cardiovascular (CV) outcomes are either neutral or adverse. In the Action to Control Cardiovascular Risk in Diabetes (ACCORD) trial, insulin intensification increased the mortality risk.

Given these findings, the U.S. Food and Drug Administration (FDA) mandated that all diabetes medications except insulin must undergo trials for CV risk if a safety signal is detected. Because metformin was launched in the United States before this mandate, no CV safety trial was required. However, the question remained: Does metformin confer any CV benefit? A recent trial by Holden et al.^1^ answered this question, demonstrating that insulin therapy with concomitant metformin reduces the risk of death and major adverse CV events compared with people treated with insulin monotherapy.

Over the past several years, new drug classes have been introduced, including glucagon-like peptide-1 receptor agonists (GLP-1 RA) and sodium-glucose co-transporter 2 inhibitors (SGLT2). Although these drugs have shown efficacy in lowering glucose, CV outcomes are dissimilar, particularly within the class of GLP-1 RA medications. For example, recent studies have shown that treatment with liraglutide^2^ and semaglutide^3^ significantly lowers rates of CV death, nonfatal myocardial infarction, and nonfatal stroke among type 2 diabetes (T2D) patients who are at a high CV risk. However, treatment with lixisenatide has shown no significant CV benefit.^4^ Empaglifloin^5^ and dapagliflozin,^6^ both SGLT2 inhibitors, have shown significant CV benefits. Part of the differences in outcomes may be attributed to the evaluated patient populations, which are often not the same in different trials. Approximately 20 clinical trials are currently underway to determine the CV risk associated with various diabetes drugs and drug combinations. Because these trials are using different populations and endpoints, it is important that clinicians review these studies carefully to choose the appropriate therapy for each patient.

#### Continuous glucose monitoring in multiple daily insulin injection and increasing use

Adoption of real-time continuous glucose monitoring (rtCGM) among individuals with type 1 diabetes (T1D) in the United States has grown significantly over the past 7 years, from 7% to 24%. Currently, only two rtCGM systems (Dexcom and Medtronic) and one professional system (Abbott Libre) are commercially available in the United States, and the Senseonics implantable rtCGM is under FDA review. Google, IBM, and Apple are also working on rtCGM technologies, developing sensors that do not require a transmitter. Based on this growth, rtCGM is likely to be “the future” for individuals with T1D and insulin-treated T2D. Medicare and Medicaid have recently approved to reimburse for rtCGM in the United States. Importantly, the FDA recently cleared one rtCGM system (Dexcom G5) for nonadjunctive use. Other than the daily calibrations, patients can dose their insulin without confirmatory testing with a blood glucose meter.

Although rtCGM has been used mainly by individuals on insulin pumps, studies have shown significant glycemic benefits associated with rtCGM use among T1D and T2D individuals on multiple daily insulin injection (MDI) therapy.^7–9^ Therefore, it is important that clinicians look at the usability of rtCGM not only in people on pump therapy but also in patients using MDI.

#### Beyond HbA1c as an indicator of glycemic control

It is known that glycated hemoglobin (HbA1c) is not a reliable indicator of glycemic control. It does not detect day-to-day glucose fluctuation or, importantly, hypoglycemia. Moreover, interferents, such as iron supplements, as well as racial differences, can influence HbA1c results. Given these limitations, the FDA may be moving beyond HbA1c to “time in range”—within, above, and below glucose targets. rtCGM facilitates making these assessments.

The FDA is also expanding its definition of hypoglycemia. In the past, hypoglycemia was defined as glucose < 70 mg/dL. However, the FDA is expected to issue new guidelines for drug trials, requiring investigators to include <60 mg/dL and < 50 mg/dL as additional cut points in their assessment of hypoglycemia.

#### AACE and ATTD consensus statements on continuous glucose monitoring

New guidelines from the American Association of Clinical Endocrinologists (AACE) regarding the use of continuous glucose monitoring (CGM) were recently published in *Endocrine Practice*. A key focus of these guidelines is to advocate for expanding reimbursement to cover clinician time spent reviewing and interpreting CGM data and advising patients during and outside of clinic visits.

Guidelines from Advanced Technologies & Treatments for Diabetes (ATTD) will also be published soon.

#### Hybrid closed-loop—artificial pancreas update

Ongoing research in the development of an artificial pancreas (AP) combines two technologies, CGM and insulin pumps, to create a system that delivers/suspends insulin infusion based on CGM data. Medtronic has developed hybrid closed-loop (HCL) systems, which suspend insulin when glucose levels drop below a specified glucose threshold (Medtronic 530G/630G) and suspend insulin on predicted hypoglycemia (Medtronic 640G). The Medtronic 670G system, an HCL system, provides both a low-predictive function and an auto-mode option that automatically adjusts the basal insulin every 6 days to maintain glucose levels within target range. Importantly, the Medtronic 670G system “auto-learns” how much insulin the patient needs. Future systems (Medtronic 690G) may eliminate the need for manual bolusing.

A recent study demonstrated that in-home use of the Medtronic 670G system by adolescents and adults increased time in target range and reduced HbA1c, hyperglycemia, and hypoglycemia compared with baseline.^10^ Results from a similar study also showed significant improvements in HbA1c and time in range at 3 months and 1 year, and severe hypoglycemia (SH) was virtually eliminated.^11^

Although the Medtronic 670G is the only commercially available HCL system, several AP developers are making progress with other systems, using different algorithms and, even, dual hormone (insulin/glucagon) infusion. However, these systems will not be available for at least 5 years. A key challenge to all developers will be accommodating the new ultra-rapid-acting insulins and other oral medications being developed. It is possible that new algorithms will be needed.

#### Conclusions

Because the newer medications (GLP-1 RA, SGLT2) are not necessarily the same in terms of CV outcomes, it is important that clinicians review the patient populations that have been evaluated in studies to select the appropriate medication for each patient. In the future, it is likely that most individuals will be treated with combination therapies, such as GLP-1 RA and insulin. In addition, many individuals with T1D will likely use an HCL system, depending on reimbursement availability. However, a key challenge will be to modify the current dosing algorithms to accommodate the new ultra-rapid-acting insulins and oral medications coming to market.

## Session A: Keynote Lecture

### Clinical Trials Revisited—DCCT/EDIC/UKPDS: What Did It Mean Back Then and What Does It Mean Today?

Simon Heller, University of Sheffield, Sheffield, United Kingdom

#### Background

Before the Diabetes Control and Complications Trial (DCCT) and the United Kingdom Prospective Diabetes Study (UKPDS), there were two schools of thought regarding control in diabetes. Cahill and colleagues believed that microvascular complications are a consequence of hyperglycemia and insulin deficiency, and that control of metabolic events is a factor in their progress;^12^ whereas Siperstein and colleagues felt that that clinicians must weigh the potential benefits of aggressive insulin therapy against the known harmful consequences, particularly hypoglycemia, given that there was no evidence demonstrating the link between good glycemic control and the development/progression of diabetic complications.^13^

#### DCCT and UKPDS review

Findings from the DCCT and UKPDS resolved the dispute between Cahill and Siperstein by demonstrating that the benefits of improved glucose control outweighed the risks associated with the side-effects of treatment (e.g., hypoglycemia). In both studies, reductions in HbA1c were associated with reductions in microvascular complications. Subsequent long-term follow-up analyses of both study cohorts also showed reductions in macrovascular complications. However, as demonstrated in the DCCT, reductions in HbA1c were linked to increased incidence of hypoglycemia. As a result of this research, the change in practice has been long-lasting. Published guidelines from many medical organizations now advocate that healthcare professionals aim for near-normal glucose levels in their patients, proposing HbA1c levels of between 6.5% and 7% for both T1D and T2D. However, these organizations also emphasize the need to individualize glucose targets to each patient's needs and clinical status.

#### Impact of intensive versus conventional glucose-lowering therapy in diabetes

Rates of microvascular disease have fallen and many patients live longer and healthier lives without severe complications, such as blindness, amputation, and dialysis. For example, in 2010, the relative risk of myocardial infarction was 1.8 compared with 3.8 in 1990. Similar improvements have been seen in stroke, lower-extremity amputation, and end-stage renal disease. However, questions remain as to whether glycemic control, alone, or other interventions are impacting these endpoints. Studies, such as the ADVANCE ON,^14^ a 5.9-year follow-up to the ACCORD trial, showed no improvement in CV outcomes with intensive glucose control. Moreover, as shown in the STENO 2 study,^15^ use of intensive interventions with multiple drug combinations and behavior modification conferred sustained beneficial effects with respect to vascular complications and on rates of death from any cause and from CV causes. However, a significantly greater number of patients achieved their treatment goals for blood pressure and cholesterol; the percentage of intensively managed patients who achieved their HbA1c goals was less than 20%, which was not significantly different from control patients. This suggests that interventions aimed at lowering blood pressure and cholesterol levels had a greater impact on macrovascular outcomes than glycemic control.

#### Developing more effective models of care

Despite strong evidence for the benefits of tight glycemic control, most people with diabetes continue to struggle to achieve and maintain the glycemic levels that are necessary to prevent diabetic complications. There are several reasons for this.

First, our models of care may be inappropriate. For example, a recent meta-analysis of diabetes treatment strategies identified several approaches that have been shown to improve glycemic control.^16^ Among the most effective ones were promotion of self-management, use of multidisciplinary teams, case management, and patient education. What was not tested in this meta-analysis is whether combining these approaches would be even more effective in helping patients reach their glycemic targets.

Second, there is a continued failure by healthcare professionals to recognize and acknowledge that the major determinant of outcomes is not clinician competency. In my view, successful treatment is dependent on our ability to help patients overcome their unique obstacles to effective self-management. Unfortunately, we tend to ignore the significant demands that effective diabetes management makes on patients.

Third, I believe we are struggling to achieve unrealistic HbA1c goals, which set up both our patients and ourselves to fail. This is a strong reason for individualizing glycemic targets and therapies and being more realistic in agreeing targets, particularly in those individuals who find the onerous and relentless task of self-management beyond them. These individuals require a more sensitive approach that acknowledges this, yet allows them to receive ongoing care and continued support from the professional team.

Fourth, and perhaps most important, we fail to blend the individual elements of diabetes management into a coherent care package that we can sustain. Patients are ultimately responsible for managing their diabetes, not the healthcare professional, and the outcomes are almost entirely dependent on them and their families.

#### Technology and diabetes

Although technology has a considerable potential to facilitate more effective self-management and outcomes, we need to be much more thoughtful in how we design and implement our technology. An example of this is insulin pump therapy.

In the United Kingdom, ∼6% of individuals with T1D use an insulin pump compared with ∼40% in the United States. Although proponents of pumps suggest that more people in the United Kingdom should be offered insulin pump therapy, the benefit of pump technology is unclear because few trials have compared insulin pump versus MDI in conjunction with comparable training in insulin adjustment. To address this issue, we conducted a study (Relative Effectiveness of Pumps over MDI and Structured Education [REPOSE]) to compare the effectiveness of insulin pumps with multiple daily injections in T1D adults, with both groups receiving equivalent training in flexible insulin treatment.

#### REPOSE trial

The REPOSE trial was a 24-month, parallel-group, cluster randomized controlled study that randomized 317 subjects who were willing to undertake intensive insulin treatment but with no preference for pumps or MDI.^17^ All participants attended an established group training course that taught flexible intensive insulin treatment (Dose Adjustment for Normal Eating [DAFNE]). At 24 months, HbA1c had improved in both groups but with no significant between-group differences. SH rates also fell overall (by 50%) but with no between-group differences. Although consistent improvements in quality of life (QoL) measures were observed in both groups, HbA1c levels remained well above recommended national and international targets. We concluded that our results support a care pathway for adults with T1D that starts with initial structured training using MDI, with pumps offered later to those in whom the limitations of MDI interfere with effective self-management. Moreover, we found that structured training reduces the risk of SH and leads to modest but long-lasting benefits in HbA1c. The results also emphasize that diabetes professionals need to ensure that far more individuals with diabetes participate in structured training as a crucial part of their management package.

#### Summary

Reproducing beta cell physiology and reversing the metabolic abnormalities of diabetes has proved challenging. Moreover, we have failed to understand the crucial contribution that people with diabetes make in managing their condition and determining treatment outcomes. Knowledgeable healthcare professionals who have the time and enthusiasm to support patients in their self-management are needed. Although successful use of technologies depends on equipping patients with the requisite skills, we urgently need to develop new approaches to engage and support them in their diabetes self-management. New therapies and technological innovations offer considerable promise; however, we need to combine these tools with novel interventions, which promote effective self-care behaviors.

## Session B: Diabetes Management

### Practical Implementation of Continuous Glucose Monitoring and Insulin Pump Therapy

Jan Soupal, Charles University Prague, Prague, Czech Republic

#### Background

Long-term use of rtCGM provides significantly better clinical outcomes compared with treatments based only on self-monitoring of blood glucose (SMBG). However, to optimize use of technologies such as rtCGM and insulin pumps, physicians and patients must learn how to use them effectively. Until now, there are few recommendations for rtCGM use in basic clinical scenarios and especially in everyday life situations.

#### rtCGM alarms and target range settings

Numerous clinical trials have demonstrated that the benefits of rtCGM are seen primarily in those patients who regularly used their devices >70% of the time. Current guidelines support this level of use. However, patients are not always willing to use their rtCGM device at that level of frequency due to limitations of the technology. Therefore, to improve adherence, clinicians must find ways to remove patients' barriers to using rtCGM.

A 2011 article in *Diabetic Medicine* reports that patients consider alarms to be a significant limitation of rtCGM use.^18^ If alarm settings are too narrow, patients can be substantially disturbed by these alarms. This can lead to patients over-reacting to the alarm or, at the other extreme, patients might start ignoring the device. Both extremes can lead to frustration and alarm fatigue.

One must also consider the important clinical aspects of this issue. Patients usually react after the target glucose limit is exceeded. If the target is too wide, the result can be higher glycemic variability with more time spent in hypoglycemia and hyperglycemia. In addition, individuals who have fear of hypoglycemia may oscillate around the upper limit of their target range, resulting in chronic hyperglycemia. In essence, an alarm setting that is too benevolent may increase patients' risk of failing to meet their glucose targets.

In my clinical practice, successive narrowing of the target range is fundamental because it allows us to gradually decrease mean glycemia and improve the HbA1c. If a patient has not shown improvement in HbA1c at 3–6 months after rtCGM initiation, this is an indication that the alarm settings are too benevolent.

#### Adjusting insulin doses according to trend arrows

A key feature of rtCGM devices is the ability to display the direction and velocity of glucose change via rate of change (ROC) arrows. This information can be used to guide insulin dosage calculations and adjustments. Based on data from early studies of rtCGM use, it is commonly recommended that patients adjust their insulin dosages no more that 10%–20%, up or down, depending on the degree of glucose change, as indicated by the trend arrow. However, a recent article from Pettus and Edelman suggests that these adjustments may be too conservative.^19^ In the article, the authors propose guidelines, which are based on a survey of 222 rtCGM users with well-controlled diabetes, recommending that insulin dosage decisions should be based on the 30-min “anticipated” glucose levels indicated by the immediate ROC arrow(s). Insulin dosages are then calculated, using the glucose target and trend graph, according to the “50–75–100” rule.

For example, for a patient using the Dexcom rtCGM system, two “up” ROC arrows indicate that the 30-min anticipated glucose will be ∼100 mg/dL higher than the current glucose. The patient would then calculate the insulin dose to address the anticipated glucose level. Decisions for treatment adjustments (e.g., insulin reduction/suspension, carbohydrate intake) are similarly based on “down” ROC arrows.

#### “Appropriate” patients for permanent use of rtCGM

In a recent prospective, real-life study, we looked at the impact of various combinations of insulin delivery/glucose monitoring methods on glycemic control in 65 T1D patients.^20^ One group of patients opted to use rtCGM with either insulin pump or MDI, and they agreed to use their rtCGM device >70% of the time. The other group continued SMBG with either insulin pump or MDI. All patients were highly motivated, and patient preferences and needs were strongly considered. At 1 year, significant HbA1c improvements were seen among rtCGM users compared with SMBG users regardless of insulin delivery method. We concluded that high motivation is key to the effective use of rtCGM. However, the question is: How do we identify these patients?

In our center, we have a process for identifying patients who have the ability and willingness to respond to information from rtCGM. The first step is blinded CGM for 1 week to confirm indications for rtCGM use and to detect possible mistakes in insulin dosing. The second step rtCGM use for 1–2 weeks provides basic education about rtCGM at initiation. If patients show even minimal improvement in glycemic control, we view this as a good marker for successful long-term rtCGM use.

#### Summary

The T1D population is a diverse group of individuals, each with specific needs and lifestyles. Many of these patients will benefit from use of rtCGM and other diabetes technologies. Use of sensor-augmented insulin pumps with automatic insulin suspension may help patients restore this awareness and, thus, avoid hypoglycemia; whereas other patients may realize significant benefits from rtCGM use in combination with MDI therapy. Conversely, other patients may be unwilling to use any of these devices, especially patients who are satisfied with SMBG and are able to achieve good glycemic control. Therefore, individualizing treatment is crucial for an effective diabetes management.

## Session C: Insulin Pump Therapy

### Do Insulin Pumps Dose Accurately? A Comparative Evaluation Study

Ralph Ziegler, Diabetes Clinic for Children and Adolescents, Muenster, Germany

#### Background

There are two basic types of basic insulin pumps: durable devices and “patch” pumps. Durable devices infuse insulin via catheters and infusion sets. Patch pumps are tubeless and attach directly to the body. There are differences in specific features and setting options among all insulin pump devices.

When assessing the accuracy of insulin pumps, one must consider important factors. First, one must look at both the basal and bolus insulin delivery in terms of the actual amount of insulin infused and how fast the insulin is being delivered. The accuracy and timing of occlusion alarms must also be considered. Moreover, it is important to differentiate between true pump errors and errors in performing the actual measurement. One must also consider the statistical methods used. For example, should accuracy be measured by a single dose or the average of several doses over a specified period? Although it is commonly assumed that insulin pumps accurately deliver their dosages as programmed, few studies have looked at the accuracy of these devices.

#### Current evidence

Accuracy of insulin delivery is particularly important in younger pediatric patients because they are taking small insulin doses, especially in basal rates, and often have varying basal rates (temporary or according to circadian rhythms). A study by Borot et al. compared basal insulin flow rate accuracy among five insulin pump systems at 1.0 and 0.1 U/hr.^21^ Although significant variability was observed during the first 15 min of the assessment, all of the insulin pumps were within ±5% flow error at 4 h and remained at that level up to 24 h at an infusion rate of 1.0 U/h, when calculated over time, not for the single burst. However, the reported flow error increased significantly in some insulin pumps when the infused dose was lowered to 0.10 U. Other studies have shown similar findings.^22,23^

Although data regarding circadian or temporary basal rates are sparse, it is known that basal rate accuracy at 0.1 U is notably low with errors of up to ±30%. It has also been shown that it can take ∼2–4 h until a change in basal rate achieves steady state. Moreover, there are notable differences among insulin pumps in the ability to accurately deliver smaller increments of insulin when temporary basal rates are used. Accuracy studies in bolus insulin delivery are also sparse^24^; however, data provided by manufacturers show error rates ranging from ≤±5% at maximum bolus to ≤±50% at minimum bolus dosages.

#### Ongoing dosing accuracy study

A new study is underway to assess the dosing accuracy of six different insulin pump models, using different insulin infusion sets (IIS). Funded by an unrestricted grant from Roche Diabetes Care GmbH, the Dosing Accuracy of Different Insulin Pumps IDS Comparative Evaluation (ICE) study is being conducted in an independent, certified, and accredited laboratory, following the International Infusion Pump Standard EN60601-2-24, which provides technical details for measuring insulin dosing accuracy. Although the measurement standard provides guidance for testing accuracy, it does not provide definitive values or limits, as seen with SMBG accuracy standards. The study is investigating several key aspects of accuracy, including: bolus and basal insulin delivery at different dosages and rates (continuous and circadian rhythm); temporary basal rates; basal/bolus delivery in “real life”; and occlusion detection. Researchers from different groups will analyze and publish the final results.

Preliminary findings have shown notable differences across pump models, using different IIS. In our assessment of bolus insulin accuracy, the maximal error of the median bolus was much smaller than that of single boluses: 10.2% versus 64.0% with 0.1 U, 2.2% versus 42.0% with 1.0 U, and 1.0% versus 8.0% with 10 U. Considerable differences were observed across pump models with different IIS in the scattering of single boluses, showing significantly less accuracy with smaller insulin boluses. Notable differences in insulin delivery time and the timing of occlusion alarms have also been observed in our assessments. Results from our assessments of basal insulin infusion accuracy are not yet available.

#### Summary

Preliminary results from the ICE study, and those from earlier studies, report significant differences in insulin delivery accuracy among commercially available insulin pumps. Although the clinical relevance of insulin pump accuracy for insulin pump users, in general, has yet to be fully elucidated, we do know that these differences are important in certain populations, specifically younger pediatric patients. Moreover, one must also consider the impact of these differences as we move forward in our development of closed-loop insulin delivery systems, which rely on the accuracy of both the CGM devices and insulin pumps.

### Insulin Pump Therapy and Sports

Philippe Lysy, Cliniques Universitaires Saint-Luc, Brussels, Belgium

#### Background

Exercise is the third essential component in blood glucose regulation after insulin and dietary management in T1D. The benefits of exercise are well matched with diabetes because it reduces glucose consumption, increases insulin sensitivity, and decreases postprandial and fasting glycemia. The resulting increase of muscle mass further increases glucose consumption during exercise. Importantly, exercise decreases all-cause, CV disease-related mortality and reduces the risk of developing T2D by ∼60%. These benefits constitute per se a major argument for promoting sports in youth with diabetes because some extents of CV disease may already prevail during adolescence. However, it is recognized that moderate intensity exercise increases the risk of hypoglycemia whereas vigorous exercise is associated with secretion of counter-regulatory hormones (adrenaline and glucagon), provoking hyperglycemia and ketosis.

#### Different forms of exercise have different effects on glycemic levels

Exercise can be characterized into extreme aspects, such as duration, intensity, and metabolic features. Aerobic exercise tends to lower blood glucose, whereas mixed exercise with bursts of anaerobic activity have mixed effects on glucose levels but generally lead to decreased glucose. Conversely, anaerobic exercise tends to increase blood glucose levels, and it is sometimes recommended to patients to finish their workout with the burst of maximal exercise to reduce the risk of hypoglycemia.

#### Recommendations for physical activity

It is generally recommended that adults try to achieve 150 min/week of accumulated physical activity (PA; e.g., brisk walking) with no more than two consecutive days off, and 2–3 days/week of resistance training for muscle strength. Children aged 5–11 years should accumulate >60 min of moderate to vigorous (50%–75% VO_2_max) PA daily. Most individuals with diabetes do not meet these minimum recommendations, mainly due to fear of hypoglycemia.

#### Recommendations for insulin adjustment

A key variable of glucose control during sports is the timing of insulin administration. In this regard, insulin pump therapy has an enormous advantage over MDI because it allows for fine-tuning of insulin administration during sports. Based on accumulated evidence regarding insulin pump adaptation in sports, it has been suggested that the prandial bolus insulin be reduced 20%–50% when exercise is to be performed in the early postprandial state. For later postprandial exercise, patients may be told to disconnect their pump or set a temporary basal rate (30%–80%) at least 90 min before starting the exercise. A 50% correction bolus afterward may be needed if the exercise is intense. For optimum overnight glucose control, patients may be counseled to decrease their basal rate by 10%–20% from 9 p.m. to 3 a.m. With all-day or unusual activities (e.g., exercise camps), patients may consider a 30%–50% reduction in the basal insulin throughout the day and the night after the activity.

Although these recommendations are supported in the literature, it is difficult to establish definitive protocols for insulin adjustments during and after exercise because the planning of basal rate modifications does not always provide the expected outcomes. For example, a study by Franc et al. found that insulin pump interruption resulted in more hypoglycemic events and less hyperglycemia than an 80% reduction in basal rate for similar exercise protocols.^25^

#### Current research

We performed a study of 11 continuous subcutaneous insulin infusion (CSII)- and 13 MDI-treated pediatric patients who performed two 30-min sessions of moderate to vigorous exercise on a treadmill under CGM.^26^ The first sessions were conducted without insulin modification; the second sessions were conducted after preemptive algorithm-based insulin dose modifications. Although insulin adaptations did not modify immediate postexercise decreases in blood glucose in either group, glucose levels improved in the CSII-treated patients during the 16 h postexercise, with up to 86% of glucose levels within target. Reduced rates of hypoglycemia during 4 h postexercise and shorter duration of nocturnal hypoglycemia were also observed. Conversely, glucose levels did not normalize in MDI-treated patients, who experienced higher rates of hyperglycemia during the afternoon snack with no changes in percentage of hypoglycemia. In summary, we found that our algorithmic adaptations of insulin doses were associated with better outcomes in terms of postexercise glucose control in patients with CSII therapy but not with MDI treatment.

#### Summary

Although patients with T1D have idiosyncratic glycemic response to exercise, most of patients' sports are associated with a risk of hypoglycemia. This requires clear and written recommendations for insulin adaptation for both—patients under multiple daily injection regimen and CSII (or pump therapy). Up to now, these are either lacking or difficult to adjust individually. Further research is required for developing algorithms for insulin adaptation and consumption of carbohydrates, based on individual characteristics and on the intensity and type of PA.

### Hypoglycemia Increases Morbidity, Mortality, and Healthcare Cost

Brian Frier, University of Edinburgh, Edinburgh, United Kingdom

#### Background

Hypoglycemia is a condition in which abnormally low plasma glucose confers harmful effects on the brain, causing both neuroglycopenia and profound activation of the sympatho-adrenal system, resulting in end-organ activation and the classical symptoms of autonomic activation (e.g., tremor, pounding heart, sweating) that are typical of a classic “fight or flight” response. It also stimulates the parasympathetic nervous system, which would cause bradycardia, but this is masked by the more profound sympatho-adrenal response. Although hypoglycemia prompts the release of large amounts of catecholamines, these do not generate the symptoms; rather, they increase their magnitude, and their secretion also promotes major hemodynamic and hemorrheological changes.

#### How should hypoglycemia be defined?

In clinical use, we generally define hypoglycemia by whether an individual can treat himself/herself. Self-treatment is considered mild or non-SH, irrespective of the symptoms; whereas episodes that require external assistance for recovery are considered severe. However, reaching a consensus on the biochemical definition of hypoglycemia has been challenging.

Since 2005, the American Diabetes Association (ADA) has defined hypoglycemia as glucose ≤ 70 mg/dL (≤3.9 mmol/L), which is the cut point that has been used since then in most clinical trials. Based on recommendations from the International Hypoglycaemia Study Group, the ADA and the European Association for the Study of Diabetes (EASD) recently published a joint position statement defining SH as glucose < 54 mg/dL (3.0 mmol/L) and recommending that this cut point be used in all subsequent clinical trials.^27^ When assessed by CGM, SH is defined as < 54 mg/dL (3.0 mmol/L) lasting for at least 20 min.

Several factors support the new measures. One is that glucose < 54 mg/dL (3.0 mmol/L) indicates unequivocal hypoglycemia in nondiabetic individuals, and it approximates to the threshold at which symptoms are generated and cognitive impairment commences. It is also the level associated with the longer-term complications of hypoglycemia, defective counter-regulation, and the syndrome of impaired awareness of hypoglycemia. Moreover, the risk of developing a severe hypoglycemic event is significantly increased at this level.

#### Does hypoglycemia cause morbidity?

The morbidity associated with hypoglycemia can be categorized into three groups. The musculoskeletal effects include falls, fractures, and dislocations resulting from accidents. Insurance claims data from the United States show that hypoglycemia is significantly associated with accident risk and fall-related fractures in people aged > 65 years.

The CV effects include myocardial ischemia and cardiac arrhythmias. The sympatho-adrenal activation has a profound effect on the workload of the heart, with a rapid rise in myocardial contractility and cardiac output.

The acute neurological consequences of hypoglycemia include cognitive dysfunction, coma, and seizures, and they can precipitate hemiplegia. At glucose levels of < 54 mg/dL (3.0 mmol/L), the ability to complete tasks that are complex, attention demanding or involve speeded responses is impaired. The brain slows down, but accuracy is preserved at the expense of speed. Moreover, cognitive recovery is delayed; it takes ∼45 min or more for all the cognitive domains to return to normal. This lag time between blood glucose normalization and brain recovery is particularly dangerous (e.g., in situations in which an individual resumes activities, such as driving, after treating a hypoglycemic event). The long-term effects of recurrent hypoglycemia on the brain include impaired hypoglycemia awareness, counter-regulatory hormone deficiencies, and stroke. A key concern is cognitive decline, particularly in older individuals. Studies in this population are ongoing to determine whether recurrent exposure to hypoglycemia is accelerating cognitive decline or, possibly, inducing dementia.

In T1D, hypoglycemia-associated mortality rates are estimated at 4%–10%. The most common cause of death from hypoglycemia appears to be cardiac arrhythmia. Although hypoglycemia-related traffic accidents and other forms of misadventure are also known causes of mortality, death from neuroglycopenic brain damage is relatively uncommon.

#### What is the economic cost?

SH imposes significant costs on individuals with diabetes and healthcare systems. Because estimating the economic cost of hypoglycemia to the individual patient is difficult, surveys have focused primarily on the direct financial expense incurred by healthcare systems. This can be measured as direct and indirect costs. Direct costs include treatment consumables, costs associated with utilization of emergency ambulance services and medical staff, hospital admission, and follow-up expenses. Indirect costs assess loss of productivity and earnings.

The highest direct costs are associated with hospital admission and inpatient treatment, although the number of cases is small. Because subsequent follow-up also utilizes resources, hypoglycemic events that require the attendance of healthcare practitioners generate higher costs than those treated in the community by relatives or friends.

Although people with T2D have a lower overall risk of developing SH than those with T1D, a higher proportion requires emergency medical treatment, and because a much larger population is at risk, the absolute number of severe events is much higher. Moreover, the average cost of a severe hypoglycemic event is higher in insulin-treated T2D than in T1D.

#### Summary

SH is a significant health concern. Most importantly, SH is an avoidable complication of diabetes treatment that inflicts significant risks for morbidities and mortality on individuals with diabetes and imposes a substantial economic burden on the provision of medical care. A major gap exists in our knowledge regarding the cost of treating the morbidity resulting from hypoglycemia, much of which is not identified or reported. Many of the deaths in older people are attributed to CV causes when, in fact, hypoglycemia may be the precipitating factor.

## Session D: Continuous Glucose Monitoring

### The New Accu-Chek Insight^®^ CGM System—First Patient Experience

Guido Freckmann, Institut für Diabetes-Technologie (IDT), Forschungs- und Entwicklungsgesellschaft mbH an der Universität Ulm, Ulm, Germany

#### Background

The Accu-Chek Insight CGM system comprises the sensor, which is attached to the abdomen, and a smartphone app, which receives data from the sensor via Bluetooth technology. A working electrode functions on both sides of the sensor with multiple spots to create a large area under the skin. The electrodes are surrounded by two membranes. An inner membrane controls glucose diffusion from tissue to the electrodes. The outer membrane enhances the tissue compatibility of the implanted sensor. Calibrations are performed twice daily with a blood glucose meter via the smartphone app. The glucose data can be downloaded to the Accu-Chek^®^ Smart Pix software for analysis.

The system utilizes a highly predictive algorithm for reducing the time delay. Time delay, or “lag time,” is caused by three factors: physiologic delay, physical time delay, and algorithmic time delay. The physiologic delay refers to the time required to diffuse glucose from blood capillaries through interstitial fluid to the sensor surface. The physical time delay refers to the time it takes for the glucose to diffuse through the sensor membranes to the working electrode. The algorithmic delay is caused by filtering out “noise” from the sensor raw data.

#### First patient experience

A study that assessed user experience with the system among 40 patients with T1D in a home-use setting was conducted at the Institut für Diabetes-Technologie (IDT). The majority of patients agreed that insertion was easy to learn and perform. Most respondents reported that the insertions were associated with no or very light pain. In addition, most patients reported that handling was very easy and that the system was comfortable to wear. Respondents were especially positive about the smartphone app design and usability.

#### Accu-Chek Insight CGM system performance

In a recent study conducted at IDT, Ulm, and Profil, Neuss looked at the performance of the system under normal life conditions. The study included 36 individuals with diabetes (27 T1D, 9 T2D) and was designed according to Clinical and Laboratory Standards Institute (CLSI) guidelines (POCT05A). These guidelines recommend assessment of fast glucose dynamics and time in hyperglycemic/hypoglycemic ranges.

The sensor-to-blood glucose difference, mean absolute relative difference (MARD), and lag time between blood and interstitial glucose levels were calculated based on the study data. MARD is the average of the absolute error between all CGM values and matched reference values. Although MARD is a common accuracy metric in CGM studies, it is dependent on the glucose ROC, which, in turn, can be influenced by the study design, reference methodology, sensor wear time, and even patient group. To address part of these limitations, we assessed the precision of the sensors, calculating the percent of the absolute relative difference (PARD). PARD is a metric that is used to compare glucose readings from two identical CGM sensors, working simultaneously in the same patient. Each patient wore two CGM sensors simultaneously for 7 days. The time delay between blood glucose and interstitial glucose values was also assessed; investigators induced glucose variations on two of the seven study days.

Our analysis showed an overall MARD of 10.6%, which was consistent across all glucose ranges measured. Accuracy during the dynamic phases with induced glucose variations was similar, indicating that the time difference (lag time) between the blood glucose and interstitial glucose values was small. Our results showed an overall PARD of 9.1%; the highest PARD (12.2%) was observed in ≤ 70 mg/dL glucose range. A 44.4% reduction in the time delay was observed: from 9 min (with raw data) to 5 min (with processed data).

#### Status of the system

The system received a CE mark at the end of 2016 and was launched in The Netherlands, Sweden, Norway, and Denmark in early 2017 via specialized diabetes centers. Roche Diabetes Care is in close contact with patients to obtain additional information on usability and acceptance. This feedback will be used for further product improvements.

### Get the Big Picture—Managing CGM Data with the Accu-Chek Smart Pix Software

Timm Wiedemann, Roche Diabetes Care GmbH, Mannheim, Germany

#### Overview

The Accu-Chek Smart Pix system was introduced in 2005 with a device reader that uploaded blood glucose data from infra-red Accu-Chek meters for visualization of glucose data profiles and statistics. This was followed by the Accu-Chek Smart Pix device reader (version 2.0) for infrared- and Universal Serial Bus (USB) devices. In 2008, Roche Diabetes Care launched the Accu-Chek Smart Pix software, which supports all Accu-Chek devices (blood glucose meters and insulin pumps) and is widely accepted in the market.

#### Accu-Chek Smart Pix software (version 3.0)

The newest version of the software (version 3.0) was launched in March 2017. The software now features an enhanced user interface that is customizable for basic and advanced needs. Importantly, the software supports the Accu-Chek Insight CGM system and features a generic import capability that allows for integration of data from the Eversense^®^ CGM system (Senseonics, Inc., Germantown, MD). Reports (e.g., trend, standard day/week graphs) and statistics have been adapted to CGM data presentation. In addition, the ambulatory glucose profile (AGP) view for data standardization has been included. CGM day clustering and mealtime-related minimum alignment features have also been added.

#### Reports and features

The *Status* screen provides an overview of patients' CGM and insulin data. The data are automatically interpreted, showing the hypoglycemia duration, median glucose, and glycemic variability along with tracings of all CGM days covered. This page is the starting point for analysis/interpretation, with links to all of the other pages.

The *Standard Day/AGP* screen presents a visualization of a standard day, which includes all CGM readings according to percentiles—10%, 25%, 50%, 75%, and 90%. We also added a minimum line that identifies the lowest glucose point of all days to visualize areas when hypoglycemia occurred. The software also allows the user to identify a specific point and obtain additional information (e.g., day, date, glucose value) about the event. The data can also be viewed in time blocks for mealtimes and overnight, which can be individualized.

The software features an algorithm that compares days to detect specific patterns of glycemic control and then organizes similar days into clusters. The clusters are color-coded and can be viewed in various visualization formats (e.g., tracings, calendar). Individual days and time blocks within each cluster can be analyzed. For mealtimes, the lowest glucose level is detected and interpreted as the time of carb intake. With the alignment of the supposed carb intakes, a profile of meal-related glucose courses can be presented for a meal-specific analysis of the physiological reaction.

#### Summary

The new Accu-Chek Smart Pix software builds on previous versions to support comprehensive analysis of CGM data, featuring automatic pattern detection and easier meal detection and interpretation. Because the software was recently launched, feedback from the market is not yet available.

### It's Time to Move Beyond HbA1c Toward CGM-Based Outcome Parameters

Marc Breton, University of Virginia, Charlottesville, Virginia

#### Background

HbA1c testing measures the glycation of hemoglobin in red blood cells and is the established clinical metric used to assess glycemic control, providing an indication of average glycemia over 2–3 months. HbA1c testing is readily accessible to clinicians and, because it involves a blood draw, it is not dependent on patient compliance. HbA1c was shown to be associated with diabetes complications in early trials (DCCT and UKPDS) and is used to determine and adjust pharmacologic and behavioral therapies.

The development and growing adoption of CGM technology has significantly improved our understanding of glucose fluctuations in diabetes. Through frequent and accurate representation of the prevailing glucose concentration, CGM facilitates a reliable characterization of a patient's glucose profile, from average glucose to a precise assessment of prandial excursions, hypoglycemia exposures, and, more generally, glucose variability.

#### Limitations of HbA1c

Although HbA1c provides a surrogate marker for the development of long-term complications, it does not directly measure glycemia. In addition, test results may be influenced by nonglucose-related factors, such as genetic variants, elevated fetal hemoglobin, and chemically modified derivatives of hemoglobin (e.g., carbamylated hemoglobin in patients with renal failure). Moreover, HbA1c does not reflect intra- and inter-day glycemic excursions that may lead to acute events (e.g., hypoglycemia) or postprandial hyperglycemia, which has been linked to microvascular and macrovascular complications.

#### Estimated HbA1c

Use of estimated HbA1c (eA1c), utilizing glucose values, provides a direct measurement of average glycemia. Investigators have explored different approaches to calculating eA1c, using patient SMBG data.^28,29^ However, a key limitation of many of these approaches is that several glucose values are needed for accurate calculation, something that patients are often resistant to provide. To address this obstacle, we constructed a dynamic model that creates a daily glucose profile from unstructured SMBG data (once daily SMBG), and we then used the mean glucose from 2-day parts: early day and evening. Validation studies in T1D and T2D patients showed that this approach achieves highly accurate tracking of average glycemia over time.^30,31^ A similar modeling approach can be used with CGM data.

#### Risk analysis of blood glucose data

Assessment of risk from blood glucose data can be challenging due to the asymmetry of data distribution across the glucose scale used to assess hyperglycemia and hypoglycemia. For example, 300 mg/dL would generally be considered hyperglycemic; whereas 50 mg/dL would be considered hypoglycemic. If the glucose target is 100 mg/dL, the distance between good control and hyperglycemia is four times larger than the distance between good control and hypoglycemia. This asymmetry makes use of metrics that assess excessive glycemic variability (e.g., coefficient of variation, standard deviation) as problematic. However, by transforming the glucose scale, we could address this issue. Using this approach, we determined that a blood glucose value of 70 mg/dL has an equivalent risk to a blood glucose value of 180 mg/dL. This modeling is the basis for the low blood glucose index (LBGI) and high blood glucose index (HBGI).

#### Summary

Because HbA1c testing requires only a blood draw in the clinical setting, it does not depend on patient compliance. However, it has notable limitations in terms of reliability and clinical utility. Using calculations of eA1c, average glycemia can now be determined from routine blood glucose monitoring with less than two measurements a day. Calibrated eA1c values follow the old National Glycohemoglobin Standardization Program (NGSP) standard (> 95% within 1% HbA1c of reference) and are close to the current NGSP standard (∼80% instead of 92.5% within 6% of reference value). As such, eA1c may be used within existing clinical guidelines. However, exposure to both hypoglycemia and hyperglycemia can be derived only from direct glycemic measures. Although use of either CGM itself or increased SMBG frequency can impact glycemic control, CGM can be leveraged to provide a richer representation of glucose control, utilizing fasting blood glucose, eA1c, LBGI/HBGI, and percentage of time spent within, above, and below glucose target ranges. Although these assessments may benefit from links to previous techniques (such as HbA1c), they could eventually replace them. However, clinical guidelines or normative data for CGM-derived outcomes are currently not available.

### An Implantable Sensor for CGM—First Clinical Experience

Jort Kropff, University of Amsterdam, Amsterdam, the Netherlands

#### Background

The Eversense CGM system (Senseonics, Inc.) consists of: an implantable, fluorescence-based, cylindrical glucose sensor (3.3 × 15.7 mm); a smart transmitter; and a Mobile Medical Application (MMA) that displays glucose information and operates on a mobile device that allows users to review current and historical glucose data in real time. The battery-powered transmitter is worn externally over the sensor and continuously transfers glucose data to the MMA via a secured low-energy Bluetooth transmission. The transmitter also provides on-body vibrations that alert users of immediate and impending hypoglycemia and hyperglycemia.

#### Clinical study

We studied the CGM system in 71 participants, aged ≥18 years, with T1D and T2D in a 180-day multinational, multicenter, pivotal trial. The purpose of the study was to determine the accuracy and safety of the system. The average age of study patients was 41.7 years, 59% were male, and the average duration of diabetes was 22.2 years. Mean baseline HbA1c was 7.5%. At the sensor insertion visit, a CGM sensor (in unblinded mode) was implanted in both upper arms of each participant. Patients used the CGM system at home and in clinic and were asked to calibrate twice a day. CGM accuracy was assessed during eight in-clinic visits.

Study results showed an overall MARD of 11.6% across the full glucose range (40–400 mg/dL). However, performance of the CGM system in the hypoglycemic range (≤ 75 mg/dL) was less than the overall performance, 21.7%. A Kaplan-Meier analysis for sensor survival estimated sensor functionality at days 45 (100%), 90 (82%), 135 (59%), and 180 (40%), showing a median sensor life of 149 days (IQR 97, 180). Accuracy was stable over all successive 30-day periods. Adherence was extremely high; median daily usage was 23.5 h. Subanalyses of uncontrolled data in 55 patients revealed a 0.3% reduction in HbA1c at study end, *p* < 0.001. Moreover, we saw a 20% reduction in the percentage of time spent in hypoglycemia, from 5.3% at baseline to 4.4%, *p* = 0.003.

Importantly, no severe procedure- or device-related serious adverse events were observed. Fourteen device- or procedure-related nonsevere adverse events occurred in 11 out of 71 patients with a total number of 147 sensors implanted, used, and removed. Five cases of skin reaction were recorded but, in all cases, sensor use was continued after a temporary stop of 1–3 weeks. Two cases of incision site infection occurred. One patient received antibiotic treatment; the other infection resolved without antibiotic treatment.

Preliminary data on patients' perceptions of the system showed a notable level of satisfaction with the system. Sensor accuracy, the utility of the alarms/alerts, and the ability to remove and replace the transmitter were highly scored. In addition, 90% of patients reported that they did not feel the sensor; 86% reported that the sensor insertion/removal procedure was painless; and 84% reported that they would want to continue using the system. A less favorable characteristic was the look and feel of the transmitter. However, developers are working on a second-generation system, which will feature a smaller, slimmer transmitter.

#### Improved algorithm

The data from the PRECISE trial was re-analyzed, and the results were used to modify the data algorithm to further improve accuracy. Results from a pediatric study in Europe and a large study in the United States show improved accuracy (∼10% MARD) over the full glycemic range.

#### Conclusions

The results from our study demonstrate that use of the Eversense implantable CGM sensor is both effective and safe, and it provides specific usability benefits. Our results support the viability of implantable CGM as an alternative to current transcutaneous CGM.

## Session E: There Is No One Size Fits All

### Health Disparities Between Countries and People

Edwin Gale, University of Bristol, Bristol, United Kingdom

#### Background

We live in a divided world. In fiscal terms, per capita gross domestic product ranges from $85,000/year in Singapore to $56,000 in the United States and ∼$700 in the Central African Republic. There are also major social, cultural, and gender divides within individual environments. Even the richest countries are challenged by mounting healthcare costs, and 27 million Americans are currently denied health insurance. Further, the gap between the “best” and the “rest” of healthcare is steadily widening, partly because of increasing income inequality and partly because of increasingly expensive high-tech medical solutions backed by sophisticated marketing.

#### A rising prevalence of diabetes is good for you

Prevalence is a balance between the incidence of new T2D cases and the number of people dying, and incidence is driven by risk factors, notably the rise in obesity. Today, we consume two to three times as many food calories as our ancestors. One of the consequences of this excessive food consumption has been deposition of adipose tissue centrally, and this has resulted in what people often refer to as the metabolic syndrome. I prefer to think of it as the “affluent phenotype,” one that not only carries an increased risk of hypertension, hyperlipidemia, atheroma, and diabetes but is also a factor in ∼40% of all cancers. Excess calorie intake is a major driver of the affluent phenotype, but reduced calorie intake reverses most of its features. Although the human phenotype has changed, diabetes has not.

Increasing age is another factor driving diabetes prevalence. The increasing longevity in the population worldwide is a major factor in the rise of diabetes, and it has changed the phenotype of diabetes. Old people with diabetes have different characteristics, different risks, and need different approaches to treatment. Earlier diagnosis and increased screening are other factors in the growing diabetes prevalence.

Although counter-intuitive, the rising prevalence of diabetes could be viewed as a marker of improved living conditions in healthcare. People have more to eat, are being diagnosed earlier, and are living longer after the diagnosis of diabetes. However, this is not the case for many people. Although diabetes was once considered a “Western” disease, it is now a global concern that affects many people who live in impoverished communities where adequate healthcare resources are scarce.

#### The costs of inequality

It was recently stated that the 62 richest people in the world are worth the same as 50% of the global population. Importantly, 33 of the 38 most highly indebted countries are in Africa, where average annual incomes are considerably less than $3000. With few exceptions, no one in Africa is likely to live beyond age 65. These findings demonstrate a clear relationship between income, affluence of the country, and the health of its population. There are also huge urban/rural differences in many parts of the world, based on income, affluence, and circumstance. In many parts of the world, urban life expectancy is significantly greater than in rural areas.

#### The price of healthcare

Healthcare costs, worldwide, are rising significantly. In the United States, prescription drug costs are a major contributor. IMS data show that the U.S. economy grew by 57% from 1990 to 2006 but drug costs expanded by 573% during the same period. It is clear that rising drug costs in all branches of medicine are becoming part of the health burden. According to the ADA, the estimated direct cost of diabetes in the United States was $176 billion in 2012.^32^ Most of the cost was for in-patient hospitalizations (43%) and treatment of complications (18%), followed by diabetes medications and supplies (12%). Earlier U.S. data show that ∼25% of people who are 65 years old were not covered by insurance for their medications. However, the impact of out-of-pocket medication costs extends beyond patients' adherence to medication regimes. Many respondents reported cutting back on basic needs to pay for their medications. Many others incurred credit card debt or borrowed money from family or friends to pay for their medications.

In the poorer parts of the world, medication costs are much more of a burden, relatively speaking, than they are in the developed countries. Medications are estimated to account for ∼60% of healthcare costs, and typically, 90% of these costs are out-of-pocket. Clearly, the cost of medication is a critical factor in the future health of diabetes in people on this planet.

We seem to be in a situation of diminishing returns. Years ago, we were achieving 90% of the glucose control that we are achieving now, when all we had were animal insulins, sulfonylureas, and metformin. Although the new medications have demonstrated some important benefits, in terms of just keeping people alive and in reasonable care of their diabetes, the impact has been relatively marginal.

The high and rising cost of diabetes medications, devices, and diabetes supplies is not simply a matter of manufacturers raising prices; it is also a matter of the supply chain from which various groups take their “little chunk” of the profit all along the way until it reaches the consumer.

#### Cost-effective care for diabetes

Diabetes care does not have to be expensive; it can be managed with cheap insulins and oral agents if they are available; however, ensuring availability is the challenge. Even in Africa, where sulfonylureas and metformin are the basis of people's therapy, these medications are often unavailable because the pharmacies only make money if they stock the more expensive medications.

#### Conclusions

At a practical level, the duty of every physician is to offer each patient the best possible care within the resources available. In resource-limited environments, there is the further duty to ensure that limited family or healthcare funds are used in the most cost-effective manner. A particular problem in cash-poor environments is that treatment guidelines developed in affluent societies are largely irrelevant to the majority of people worldwide who have diabetes. We need a “rough” guide for diabetes because the best possible care is not affordable for most people. We need to communicate the message that solid, evidence-based diabetes care is available at a cheap price. That message is not getting out, it is certainly not being practiced around the world, and I think that many people are suffering the consequences.

### Lessons Learned from Phenotypes in Prediabetes and Type 2 Diabetes

Norbert Stefan, University of Tübingen, Tübingen, Germany

#### Background

The prevalence of prediabetes and T2D is increasing worldwide. Prediabetes is associated with an increased risk of T2D, cardiovascular diseases (CVD), dementia, and cancer and has recently gained much attention in the field of clinical research. In prediabetes, lifestyle and pharmacological intervention can prevent diabetes and possibly CVD. Thus, the implementation of interventions in this condition is of major importance. However, prediabetes is a very heterogeneous metabolic state, in respect to both its pathogenesis and the prediction of diseases. Thus, a better understanding of its pathophysiology and stratification of the risk is needed.

#### Strategies for detecting risk

Various phenotyping strategies for identifying subpopulations at high risk for developing T2D diabetes are being explored. Studies assessing the relationship between body mass index (BMI) and elevated fasting and 2-h postprandial glucose have shown significant variability, which indicates that stratification according to BMI is likely not useful. However, we do see a distinct relationship between insulin secretion and insulin sensitivity among healthy individuals, those with impaired fasting glucose/glucose tolerance and clinical T2D.

Genome-wide association studies have identified several genes that are specific to insulin secretion, particularly TCF7L2. Although these genes appear to be highly predictive in assessing the risk of T2D development, currently they cannot be used in clinical diagnosis or in the prediction of effectiveness of the treatment due to cost and complexity.

Estimation of body fat composition and ectopic fat deposition is another approach to risk detection. However, measurements of waist circumference and waist-to-hip ratio do not differentiate between intra-abdominal and subcutaneous fat, which confer different levels of risk. This assessment can only be performed by using magnetic resonance imaging (MRI), which is expensive. Stratification of individuals with prediabetes at baseline into a high-risk and a low-risk phenotype, based on corrected insulin secretion and insulin-resistant nonalcoholic fatty liver disease (NAFLD) through ultrasound, may be helpful in determining the effectiveness of lifestyle interventions to revert individuals to normal glucose regulation.

#### Nonalcoholic fatty liver disease

Research into NAFLD has revealed important aspects for the prediction and prevention of cardiometabolic diseases. The liver plays a role in increased glucose production and dysregulated lipoprotein metabolism, conditions that are often found in patients with NAFLD. In addition, several proteins that are exclusively or predominantly secreted from the liver are now known to directly affect glucose and lipid metabolism. In comparison to the functional proteins released from adipose tissue and skeletal muscle adipokines and myokines, these liver-derived proteins are known as hepatokines.

The first hepatokine that has been proved to have a major pathogenetic role in metabolic diseases is α2-HS-glycoprotein (fetuin-A). Production of this glycoprotein is increased in steatotic and inflamed liver but not in expanded and dysregulated adipose tissue. Thus, research into this molecule and other hepatokines is expected to aid in differentiating between the contribution of liver and those of skeletal muscle and adipose tissue, to the pathogenesis of T2D and CVD. Because the diagnosis of NAFLD by liver biopsy, the gold standard method, is invasive, it is not feasible in routine practice. Consequently, there has been intense interest in blood markers that, alone or in combination with clinical parameters, would be able to identify patients with NAFLD.

#### Treatment

The most effective and safe treatment strategy to reduce liver fat content and improve hepatic inflammation and fibrosis in subjects with NAFLD is lifestyle intervention. However, many patients are not compliant with the respective recommendations. Or, in some cases, liver fat content and/or liver pathology does not improve even with weight loss. In this respect, novel studies are exploring specific pharmacological treatment approaches that may be effective and relatively safe to treat NAFLD.

#### Summary

It is well recognized that prediabetes and T2D are associated with cardiometabolic disease. Although lifestyle interventions are generally effective in treating cardiometabolic disorders, some patients may not respond. This may be due to the heterogeneity of the conditions. Precise phenotyping may help to stratify subjects with prediabetes and T2D into different at-risk subgroups so that prevention can be tailored to the major pathomechanisms and, thus, be more effective.

## Session F: Keynote Lecture

### Exercise Is Medicine in the 21st Century—Fitness Versus Fatness

Carl “Chip” J. Lavie, Ochsner Medical Center, New Orleans, Louisiana

#### Overview

Substantial data indicate the powerful impact of PA, exercise training (ET), and cardiorespiratory fitness (CRF) on long-term prognosis. Despite this evidence, PA levels remain low for most people in the United States and Westernized world. Although many factors contribute to the obesity epidemic in the United States and worldwide, my colleagues and I have demonstrated that progressive reductions in PA over five decades is the major culprit, which not only contributes to obesity but also contributes to low CRF.

#### Impact of CRF

CRF is assessed by measurement of metabolic equivalents (METs) during PA. Numerous studies have shown that higher MET levels (>10) are associated with increased longevity.^33,34^ A meta-analysis by Kodama et al. reported that low CRF or low estimated METs significantly increased all-cause mortality and CVD events, and with every one MET change, mortality and CVD events were affected by 13% and 15%, respectively.^34^ METs <7.9 were associated with poor prognosis; whereas METs >10.8 METs represented a good prognosis. Low CRF is directly attributable to low levels of PA.^35^

#### Decline in PA

PA has declined significantly over the past five decades.^36,37^

A study by Church et al., who analyzed data from the U.S. National Health and Nutrition Examination Surveys (NHANES), reported that daily occupation-related energy expenditure has decreased by more than 100 calories, and that this reduction accounts for a significant portion of the increase in mean U.S. body weights among women and men.^36^ Similar reductions in energy expenditures in women's studies have also been reported.^37^

#### “Obesity Paradox” and CVD

Although obesity has been implicated as one of the major risk factors for most CV diseases, evidence from studies of subjects with established CVD indicates an “obesity paradox”; overweight and obese individuals with these diseases tend to have a more favorable short- and long-term prognosis than thinner patients with the same CVD risks. Subsequent studies looking at the relationship between CVD outcomes and CRF have resolved this apparent paradox. A meta-analysis of 10 studies by Barry et al. found that moderately fit overweight and obese individuals had similar mortality risks as moderately fit individuals with normal weight.^38^ Other studies have shown similar findings, linking moderate levels of fitness with reductions in coronary heart disease^39^ and heart failure,^40^ regardless of BMI.

#### CFR and psychological distress

It is well known that psychological distress (anxiety, hostility, and, especially, depression) increases mortality risk. In a 2006 study, we looked at the effects of exercise on psychological distress among 635 individuals with coronary artery disease who were attending a formal cardiac rehabilitation program. Patients were assessed for depression, anxiety, and hostility, which were found to be much higher in patients <55 years (23%, 28%, and 13%, respectively) compared with patients >70 years. At the clinic, all patients were asked to exercise two to three times/week. At 12 weeks, we observed significant reductions in the prevalence of psychological distress among both study groups, but the most marked improvements were seen among the younger patients, with reductions in depression (82%), anxiety (68%), and hostility (54%). This finding is particularly important because depressed coronary patients have four to five times higher mortality during the 3 years after an event compared with those who were not depressed.

#### Low versus high doses of exercise

Although it would be ideal to elevate all patients to their highest level of fitness, this may not be necessary. Our real focus should be on improving their fitness. For example, Wen et al. looked at the benefits of leisure-time PA in a cohort of 416,175 individuals to determine whether exercise less than the recommended 150 min a week improves life expectancy.^41^ Investigators found that subjects who exercised at moderate intensity (e.g., slow walking) for ∼15 min/day had a 14% reduced risk of all-cause mortality and a 3 year longer life expectancy compared with individuals who were inactive. Every additional 15 min of daily exercise beyond the minimum amount of 15 min a day further reduced all-cause mortality by 4% and all-cancer mortality by 1%.

In a more recent study, we found that running 5–10 min/day at slow speeds (< 6 miles/h) is associated with reductions in mortality from all causes and CV disease.^42^ Importantly, when we looked at the exercise times and intensity in our study, we showed that runners in the lowest quintile (one to two times/week, < 52 min/week, < 10 km/week) experienced the maximal benefits for mortality and CV mortality, which were equal to the effects seen in quintiles 2, 3, and 4, and there was a trend toward a slightly better effect than quintile five.

However, questions regarding the benefits of intensive exercise are emerging, particularly among marathon runners and triathletes. Immediately after these events, approximately one third of athletes experience dilation of the right atrium and right ventricle, reductions in right ventricular function and ventricular septum function, and release of cardiac troponin and brain natriuretic peptide. Although these conditions resolve after ∼1 week, this physiologic response could be problematic for athletes who compete several times each year.

#### Summary

Current U.S. national guidelines recommend ∼150 min/week of moderate PA or 75 min/week of more vigorous PA. The Institute of Medicine recommends ∼60 min of total PA per day. However, recent evidence suggests that the benefits of exercise of just 15 min a day (< 90 min/week) occur even at levels well below these national recommendations. In almost everyone, it appears that the maximal benefits occur at levels of ∼40 min/day. Clinicians and public health officials should focus on PA and fitness-based interventions rather than on weight-loss driven approaches to reduce mortality risk. The promotion of PA and ET is needed throughout the healthcare system. We need to recommend PA and exercise to our patients.

## Session G: Keynote Lecture

### Diabetes Therapy in 2025

Nick Oliver, Imperial College London, London, United Kingdom

#### Background

As the prevalence, demographics, and characteristics of the global population with diabetes grow and change, the challenges to healthcare systems will evolve over the next 10 years. There will be increased incidence and prevalence of diabetes and increasing obesity, and people with diabetes will be older and live longer, creating a greater requirement for care and an increasing economic and personal burden. Solutions are required to meet these challenges.

#### Diagnosis

Diagnosing diabetes is a straightforward numerical process, based on the glucose threshold above which microvascular complications arise from diabetes. Our challenge in 2025 is not in diagnosing diabetes but in assigning diabetes subtypes. With the growing obese population, we will have an increasing number of overweight people presenting with new onset T1D; the textbook definition of significant weight loss will no longer be so applicable. With increasing obesity in children, adolescents, and young adults, the incidence of T2D in those populations will also increase, blurring definitions. The impact of migration on diabetes subtype must also be considered. For example, if a person moved from South Asia to the United Kingdom, they would keep their native T1D and T2D risk, but their children would adopt the United Kingdom white European T1D diabetes risk. Our challenge is to determine how we can accurately, appropriately, and rapidly assign the subtype of diabetes at diagnosis to ensure that people receive the right treatment immediately. In addition to measuring glucose, we will likely see more routine use of other assessments, including: C-peptide; antibody status; and genetic information.

#### Monitoring

Flash glucose monitoring has changed the landscape by enabling users to monitor glucose without fingerstick blood glucose testing, except at certain times. However, the evidence base for this technology is challenging to interpret at present. Although we have not seen improvements in HbA1c with T1D or T2D, reductions in time < 70 mg/dL (< 3.9 mmol/L) have been reported.^43,44^

Real-time CGM, which has previously been assessed in mixed populations with an overrepresentation of people using insulin pump therapy, now has an evidence base in people using multiple-dose injection regimens. As shown in the DIAMOND trial, CGM empowers people using MDI regimens to achieve improved glucose outcomes in terms of HbA1c, mean glucose, and time in hypoglycemia. Therefore, not only do we have changes in the landscape of the technology that is available to us, but also we have changes in the evidence base.^7^ In addition to the Dexcom CGM devices, we currently have the Medtronic Guardian Connect, Roche's Accu-Chek Insight CGM, and Senseonics' Eversense implantable CGM systems. Next-generation sensors promise to be smaller with improved accuracy and once daily calibration or calibration free. Sensors will have longer duration (10–14 days) and will be minimally or noninvasive, with nonadjunctive applications.

#### Closed-loop insulin delivery

The Medtronic 670G system is novel in that it utilizes both the predictive low glucose suspend and automated basal rate adjustment to address hyperglycemia. However, there are some conditions to entering the automatic insulin mode that can be challenging: All temporary basal rates must be cancelled, delivery must not be suspended, carbohydrate and low- and high-glucose settings must be established, and whoever is using it must enter a blood glucose value for the last 12 min. Moreover, capillary blood glucose testing is still required and boluses must be administered manually. Nor is it eliminating the requirement for carbohydrate counting and accurate bolusing for mealtimes. Nevertheless, a nonrandomized study showed that use of the Medtronic 670G lowered the HbA1c by 0.5%.^11^

Where is closed-loop research heading? Within the next 2 years, we will have larger, longer duration home studies and data from children younger than the age of 5 years. Hopefully, we will have data on people with new onset T1D 2 years after that. These studies will not look at glucose, specifically, as the primary outcome; rather, they will assess C-peptide as the primary outcome, investigating whether automated insulin delivery can optimize control soon after diagnosis to preserve beta cell function and prolong the honeymoon period. We will also have studies in older people with T1D, which is relevant to how we will manage people with cognitive impairment toward the end of life. After that, we will have data from home studies in people with T2D. There is a strong argument that people with longstanding T2D who are insulin deficient may derive similar benefits to people with T1D from technologies such as insulin pump therapy. Importantly, we will see studies in people with impaired awareness of hypoglycemia.

Development efforts are now underway for polyhormonal systems and polypharmacy approaches. Investigators at Boston University have shown that using a bihormonal insulin and glucagon system can improve overall time in glucose target and reduce exposure to hypoglycemia. However, concerns about glucagon stability and potential toxicities may prevent this approach from coming to fruition. Use of other hormones, such as pramlintide and exenatide, in combination with insulin are also being explored. We are also looking at the use of closed-loop systems with adjunctive oral therapies, such as SGLT2 and dipeptidyl peptidase 4 (DPP-4) inhibitors.

#### Insulins

We now have ultra-rapid insulins, which have an earlier onset, higher peak of action and faster off time than traditional rapid-acting insulin analogs. The faster-off feature, specifically, is a significant advantage in closed-loop systems. Although studies of “ultra-rapid” insulin in closed-loops systems are not yet available, in silico evaluations demonstrate a higher peak of plasma insulin that wanes more quickly, which translates into reduced postprandial glucose exposure without an increase in exposure to hypoglycemia.

Other novel insulins include buccal insulin, which is already available in the United Arab Emirates and India; however, the efficacy of this formulation is unclear. Inhaled insulin (Afrezza) is also available in some countries, and we may see a resurgence of that particularly as its rapid-on/rapid-off pharmacokinetics are well suited as an adjunct at mealtimes.

#### Cellular therapies

Doug Melton's group at Harvard recently published data showing long-term glucose control in mice with normal immune systems, using encapsulated human stem cells derived from glucose-responsive, insulin-producing islet cells.^45^ This is a very early work, and this therapy will not be available by 2025. However, they are promising data.

#### Connected health

Diabetes self-management is, essentially, a data-driven exercise. We are moving to a scenario where we have connected health, using large amounts of data collected by multiple different devices and pulling this information together to include data related to glucose, insulin, macronutrient composition/intake, activity, and health trends. Availability of these data presents an opportunity to develop decision support tools that can help people manage their diabetes more effectively. Using smartphones and apps, we will be able to deliver education, lifestyle coaching, and behavior change support remotely.

In 2025, we are going to see a change in the way we use devices. For example, automated bolus advisors have been shown to improve glycemic control. However, the equation that these devices use is fixed, and it is well known that insulin requirements are not dependent solely on glucose and carbohydrate; they also depend on glucose ROC, other macronutrients (e.g., protein and fat intake), time, day, weather, stress, activity, alcohol, hormones, and a myriad of other factors that we are not routinely measuring when we think about insulin bolus dose calculation.

Currently, we have a very clear distinction between consumer and healthcare electronics. On the consumer side, we have smartphones and activity trackers. On the other side, we have a clear healthcare technology environment, which includes insulin pumps, blood glucose meters, and CGM. Over time, consumer and healthcare electronics will come together and integrate, with data moving across what used to be the line between consumer and healthcare. We will also see changes in how we interact with people with diabetes, making greater use of remote consultation techniques.

#### Summary

Where might we be in 2025? First, I think we will change how we diagnose diabetes. We will have subtype identification at diagnosis with personalized treatment. Monitoring will be calibration free, more accurate (particularly in hypoglycemia), nonadjunctive, and with longer duration sensors that are subject to less interference. Hopefully, we will have increased access to CGM, and importantly, we will move beyond HbA1c and look at times within, above, and below target ranges and other factors (e.g., glycemic variability) as our assessment outcomes. It is likely that we will be utilizing existing agents as adjunctive therapy in T1D, and we will see progress in cellular therapy. Closed-loop systems will have an evidence base with clear indications about which subpopulations stand to benefit the most. These systems may use polypharmacy in their designs, and it is highly probable that they will be integrated with decision support capabilities. Our interactions will include more remote communication, and we will have empowered diabetes communities for peer support. In summary, we are going to be delivering connected health for people with diabetes in 2025.

## Session H: Making Best Use of the Data

### Brave New Diabetes World—Merits and Dangers of the Flood of Digital Data

Marietta Stadler, King's College London, London, United Kingdom

#### Background

The digital revolution is transforming our daily life through increasing digitalization of information, mobility of data, and improved connectivity. Approximately 3 billion people can access the Internet. We are surrounded by “the Internet of Things,” engaging in “quantified self” activities, and smartphones have become ubiquitous. More than 90% of the world data have been produced in the past 2 years. The transformation brought about by the digital revolution is having a profound effect on diabetes management and diabetes care, from the perspectives of research, medical care provision, and the individual (patient, doctor).

#### Diabetes “Digital Revolution”

Both diabetes patients and clinicians have become used to monitoring diabetes therapy numbers since the onset of SMBG. Patients are used to keeping diaries but can now do this electronically. Another reason that diabetes is one of the medical areas first affected by the digital revolution is that blood glucose meters, insulin pumps, and CGM technologies have become an integral part of our day-to-day life.

#### Utilization of “Big Data”

Big diabetes data sets are derived from various downloads of electronic patient records, social media, blogs, diabetes apps, carb counting apps, pedometers, research databases, genomics, and medical insurance data. What are we going to do with these big data? We would like to research into them to learn more, could use some for clinical quality control, and they could be used commercially.

Industry has already discovered the huge potential of these data. For example, IBM Watson Health is now collaborating with Medtronic, which has access to 125 million patient days of insulin pump and CGM data. Verily and Sanofi plan to develop a patient platform. Sanofi and Google recently announced a $500 million joint venture.

However, industry participation in this area raises questions about the ethical aspects of using Big Data. Who owns these data? The patient, clinic, college, or company? Have patients agreed that their data will be used commercially, even if anonymized? Who controls the quality of industry-initiated projects? Should academic evaluation of data proceed commercially driven analysis?

#### Impact on patients

Patient empowerment has reached a new dimension through digital media and global connectivity. Patients are becoming entrepreneurs, forming peer groups, and distributing resources for networking and individualized tools for diabetes self-management. For example, one group developed a do-it-yourself instruction for “cracking” a CGM device to transfer data to a smartphone. Others are trying to develop their own home-use closed-loop systems, which is problematic. Moreover, many of the “diabetes advice” blogs are published without any quality control. There is also the issue of patient privacy. A recent study, which looked at 211 diabetes apps to determine whether they were transferring data to third parties, found that 80% were still collecting user data and ∼50% were transferring data to other parties.

#### Impact on clinicians

The availability of data downloads from patients' devices can be very helpful for the clinician and the patient; however, the flood of health data can be overwhelming, distracting, and time consuming. As clinicians, we need to be structured in how we analyze the data, and we need to be structured in our consultations with our patients. Discussing data downloads with colleagues in the form of multidisciplinary team meetings would be helpful. However, we must also look after those patients who cannot or do not want to access the new technologies but remain skilled in their current self-management methods. The Health Innovation Network in partnership with King's Health Partners has developed the Type 1 Diabetes Consultation Tool (T1C) and an accompanying user guide for healthcare professionals (https://healthinnovationnetwork.com/wp-content/uploads/2017/01/Type-1-Consultation-Tool-User-Guide.pdf) to help with a more structured consultation for our patients.

For patients who are embracing these technologies, we can provide support and guidance, specifically as it relates to the use of online resources, such as apps and blogs. The King's College Diabetes team has reviewed these online resources and apps and has developed a website that provides recommendations about the blogs and apps that are very helpful and safe to use (https://www.t1resources.uk/home/).

#### Summary

Information technology is an integral component of our lives; therefore, it is also in the clinical care and self-management of people with T1D. However, many questions remain open for critical discussion and provide food for thought: How are diabetes clinicians and their patients going to deal with the flood of digital data? How can we keep up-to-date with the digital world that our patients are involved in? Where are the opportunities and obstacles for Big Data analysis? We are in the middle of a transformation and change in our society, and this affects our diabetes life as well.

### Data and Connectivity to Improve the Work Flow for the Healthcare Professional—What Do We Have and What Is Still Missing?

Joseph Cafazzo, University Health Network, Toronto, Canada

#### Background

Patients with diabetes are capable of doing incredible things when provided the information and tools that allow them to effectively manage their diabetes. This is particularly true of patients with T2D, who have fewer data management needs and are often not even encouraged to monitor their blood glucose by their clinician. In short, we do not give patients enough opportunities or information to self-manage their diabetes.

#### Potential of apps

Intuitively, diabetes apps appear to be a logical companion to reinforce self-management tactics, provide teachable moments, and motivate the user toward improved self-care. The real opportunity, however, is when these apps are integrated into the full clinical practice of the patient's healthcare provider. Not only would the app be prescribed, but also patients would have the means to see and reflect on lab results, communicate more frequently with their clinician, educator, dietician, health coach, and other patients, all enabled by digital means.

Several hundred mobile health apps aimed at helping patients manage their diabetes are now available. However, very few patients use them for their personal management, or have even tried them. Although the reasons behind the lack of widespread use vary, a major reason is the lack of interoperability with and between diabetes devices (e.g., blood glucose meters, CGM, insulin pumps) and other apps.

#### Need for standards

All the communication going on in the world today would be impossible without internet and data communications standards. Standards for the interoperability of devices and apps in the transfer, visualization, and integration of data open the marketplace to new entrants, researchers, and companies that may look at data in a different way. For existing players, the development of these standards could create a market ecosystem of innovation, not just for existing products but also for new products that would never even be envisioned. Unfortunately, many companies are reluctant to work together to develop these standards. Although some of the reasons for this reluctance are valid, others are short-sighted.

It should be noted, however, that regulatory agencies are becoming increasingly interested in seeing standards developed, which they feel would provide greater transparency in product development and identify security issues. This is why the FDA is constantly promoting and recognizing the need for interoperability standards.

My team has been working on the Bluetooth standard with the support of Juvenile Diabetes Research Foundation (JDRF), Roche Diabetes Care, and the Helmsley Charitable Trust. This standard provides a glucose profile specifically for blood glucose meters and CGM. In addition, the Institute of Electrical and Electronics Engineers (IEEE)-11073 standard, which facilitates communication between medical, healthcare, and wellness devices and with external computer systems, is an important step forward in AP development because it opens up opportunities to mix and match components. This would allow users to select the CGM devices and insulin pumps that work best for them. Another emerging important standard is the Fast Healthcare Interoperability Resources (FHIR) standard, which facilitates pulling data back into electronic health records.

#### Progress in achieving interoperability

The Accu-Chek Connect system enables transfer of blood glucose data from the Accu-Chek Aviva blood glucose meter to a smartphone app. Because the system utilizes the Bluetooth open glucose profile standard, we could link the meter to *Bant*, an iPhone-based app that helps adolescent users easily monitor their diabetes activities and receive personalized feedback. Users can track their blood glucose, meals (through photos), step counts, and weight, and pull data from other health apps that they may be using. We conducted a pilot study at the Hospital for Sick Children, Toronto, to assess the impact of using *Bant* among 20 adolescents on their SMBG frequency. A key aspect of the study was use of “gamification,” in which routine behaviors and actions were rewarded in the form of iTunes music and apps. The pilot evaluation showed that the average daily SMBG frequency increased by 50%. A total of 161 rewards were distributed to participants and satisfaction was high, with 88% of participants stating that they would continue to use the system.^46^

#### Summary

Addressing the growing diabetes epidemic will require a holistic approach to diabetes management, incorporating mHealth technologies into clinical care. However, this will not be achieved until healthcare providers and industry accept the role of patients in their own care, and empower them through technologies that allow data interoperability, while providing the control of privacy to the patient directly.

### The Strengths and Benefits of the New-Generation Accu-Chek Diabetes Management System

Eric Pridemore, Roche Diabetes Care, Indianapolis, Indiana

#### Background

The Accu-Chek Guide system is our latest generation of electro-chemical blood glucose monitoring systems. The system is part of a new platform, utilizing new strips with a different chemistry and a new meter measurement engine compared with the Accu-Chek Aviva/Accu-Chek Performa platforms. As we set out to develop the Accu-Chek Guide system, we defined three key customer-focused goals. The top priority for the system was to provide advanced performance, which includes advanced accuracy for all sample types, as well as to provide robust performance in wide temperature, humidity and hematocrit ranges, and limited interferences. Our second goal was to ensure that each step of the testing process included features and improvements to optimize the testing process. Our third goal was to ensure that the system provides a total solution to our patients and caregivers for enhanced diabetes management.

#### Advanced performance

The current International Organization for Standardization (ISO) standard for system accuracy (ISO 15197:2013) requires that ≥ 95% of all blood glucose test results must be within ±15 mg/dL of the laboratory reference when blood glucose levels are < 100 mg/dL and within ±15% of the laboratory reference when levels are ≥ 100 mg/dL (so-called 15/15 accuracy). Our goal, however, for the Accu-Chek Guide system was to provide tighter accuracy at 10/10.

Over the past few years, there has been much discussion about the importance of accuracy of blood glucose monitoring systems, given the varying degrees of accuracy of systems on the market. We know that falsely elevated blood glucose results can impact the ability to detect hypoglycemia, as well as present the risk of over-correcting with insulin. A falsely low glucose value can result in extended hyperglycemia, leading to elevated HBA1c.

A study by Breton et al. shows the relationship between the degree of SMBG accuracy and the risk of failing to detect hypoglycemia.^47^ The probability for missing hypoglycemia when glucose is 60 mg/dL decreases from 3.5% at 15/15 accuracy to ≤1.0% at 10/10 accuracy.

The Accu-Chek Guide test strips have undergone a wide range of testing, including studies at external sites and extensive internal testing with a goal to exceed the ISO 15197:2013/EN ISO 15197 system accuracy standard and deliver even tighter accuracy. Brazg et al. have shown the Accu-Chek Guide system achieving tighter 10/10 accuracy.^48^ Further calculations of the study data have shown that 95% of results were within ±7.5 mg/dL or ±7.5% of the laboratory reference values for glucose concentrations <100 mg/dL or ≥100 mg/dL.

An additional study evaluated the accuracy of the Accu-Chek Guide system and four other commercially available meters against a clinical laboratory method. Lot-to-lot variability (4 lots of strips for each of the 100 samples analyzed on each meter) was also assessed with each of the meters tested. Results showed that the other meters displayed significant variations across test strip lots, large variations from the lab method, and large variations within each lot.

#### Best-in-class ease of use

In developing the Accu-Chek Guide system, our goal was to ensure that each component of the system was designed to optimize the testing experience to ultimately support successful diabetes self-management. To accomplish this, we relied heavily on input directly from patients, who identified several obstacles associated with other SMBG systems: difficulty removing one test strip at a time from strip vial; accidentally spilling strips when opening the vial; difficulty seeing where to apply blood to the strip; and not being able to test in low-light conditions.

To address patients' concerns with strip and vial handling, we introduced a new spill-resistant vial. The vial holds the strip tightly in place to allow easy removal of just one strip at a time and reduces the risk of spilled strips. In a large human factors study (*n* = 197) conducted in the United States and France, 88% of patients agreed that it was easier to remove a strip by using the Accu-Chek Guide vial compared with a standard round vial.

To address difficulties with test strip dosing, we designed the test strip to allow application of the blood sample anywhere along the edge of the strip. Our human factors studies showed that this design was also rated highly by patients. We have also included a backlit display and a strip port light to help our patients test in low-light conditions.

These improvements in the testing process are designed to simplify the testing process. Given that personalized diabetes management relies on blood glucose values, we need to do everything we can to remove the hassles of testing so that patients will follow their testing regimen.

#### Enhanced diabetes management

To offer flexibility for our patients and healthcare professionals, we have incorporated key diabetes management features into the meter and the ability to transfer data to a smartphone app (via Bluetooth and USB connectivity) for automatic upload to web and desktop solutions. On the blood glucose meter, we offer the option to display the previous result along with the current result so that the patient can quickly see change from the last result. The target percentage chart shows a snapshot of percentage of test results above, within, or below target; results can be filtered by different test markers such as before meals. The meter also offers pattern detection to identify high and low patterns over the past 7 days.

Incomplete or inaccurate blood glucose data often hinder clinicians' ability to utilize SMBG data. To address this issue, the meter features low-energy Bluetooth technology to provide seamless connectivity to smartphone apps and web solutions. With the Accu-Chek Connect mobile app, patients must no longer rely on paper logbooks for documenting test results. They can also attach meal photos to their data to get assistance from their healthcare professional regarding carbohydrate estimates. The app also includes a bolus advisor for calculating insulin doses. Another important feature is the ability to automatically send glucose data to caregivers via text messages. The Accu-Chek Guide is also compatible with the Accu-Chek SmartPix desktop solution and emminens^®^ eConecta^®^, a comprehensive diabetes management solution for patients and their provider network.

#### Summary

The Accu-Chek Guide blood glucose monitoring system is designed to meet the changing and diverse needs of people with diabetes, their caregivers and healthcare professionals. The combination of advanced accuracy, easy-to-use features, and enhanced diabetes management functionality is intended to provide an innovative and highly reliable solution for daily diabetes management.

## Session I: Behavioral Change and Personalized Diabetes Management

### Toward a Healthier Lifestyle—Managing Prediabetes and Diabetes Through KALMOD

Katharine Barnard, University of Southampton, Southampton, United Kingdom

#### Background

Diabetes is a major burden for people living with the condition, for public health and for healthcare systems across the world. Despite the availability of numerous medications and medical devices to support optimal diabetes management, only a minority of individuals with diabetes reach their therapy targets. For individuals, suboptimal diabetes control has severe consequences in terms of poorer health and QoL outcomes. For healthcare systems and broader public health, the consequences are increased direct costs, days lost to work, and lack of productivity with associated financial loss to economies.

#### Scope of the problem

A key concern within the healthcare community is that many individuals with diabetes seem to not understand or appreciate the severity of the consequences of uncontrolled diabetes. Although it is well established that suboptimal glycemic control can lead to devastating clinical outcomes for people with diabetes, it also imposes a significant burden on healthcare systems. In the United Kingdom, diabetes costs the National Health Service (NHS) €11.6 billion/year, and these costs are expected to almost double by 2035. Treating diabetes complications currently accounts for ∼80% of these costs.

However, the public health burden and financial costs pale in comparison to the burden that diabetes places on each individual in their everyday living. Many people with diabetes have elevated rates of anxiety and depression, poor psychosocial functioning, poor QoL, poor well-being, negatively impacted relationships within the families, and lack of spontaneity in their lives. Combined, these factors have a negative impact on social and work activities, for both the individuals and their families.

Although patients' lack of motivation is often cited as a reason for suboptimal diabetes management, a lack of trained healthcare professionals, lack of time, and difficulty obtaining reimbursement for the most appropriate medications and diabetes technologies also contribute greatly to patients' ability to achieve and maintain optimal diabetes control. Clearly, we need a paradigm shift away from our current medical model of healthcare to a more holistic model. KALMOD addresses this need, offering a decision support system that is designed around the patient's perspective.

#### KALMOD

Based on the Kaleidoscope Model of Care,^49^ KALMOD is based on a decision-tree questionnaire that enhances patient-HCP consultations by identifying patient priorities and facilitating the development of treatment strategies that address the individual needs of each patient. It fosters a collaborative approach between the person with diabetes and their HCP and supports holistic, person-centered healthcare by identifying and articulating barriers to optimal diabetes control. This helps healthcare professionals better understand the individual needs of their patients, which, in turn, allows them to match the right patient to the right therapy at the right time. The purpose of KALMOD is to enhance the healthcare experience and improve clinical and psychosocial outcomes.

The KALMOD process involves first assessing the patient's situation. This includes documenting their personal history (e.g., socioeconomic situation, available resources) and medical history. A brief questionnaire is administered to identify individual priorities by obtaining information about his/her health beliefs, level of motivation, self-efficacy, and feeling of empowerment. Responses are translated into the basis for goal-focused, collaborative discussion presenting both priorities and care pathway options for discussion and agreement of an action plan.

#### Summary

KALMOD provides a quick and easy-to-use personalized “package of care,” which significantly improves the healthcare experience for the patient and the healthcare team.

We believe that it will streamline healthcare delivery in consultations and remove the pressure from healthcare professionals to provide “the answer” without having the required information regarding the patient's personal barriers to self-management. In addition, it matches treatment options/devices that are sustainable in the long term, which creates greater economic efficiencies for healthcare systems (e.g., minimizes discontinuation of insulin pump and CGM use). Importantly, KALMOD is scalable and modifiable to other chronic conditions. Clinical trials are currently underway, and preliminary data show that KALMOD is acceptable, relevant, and tailored to individual needs, and that it can be implemented in clinical practices.

### How to Use Data and Technology to Change Behaviors

Michael Fergusson, Ayogo Health, Inc., Vancouver, Canada

#### Overview

Patient nonadherence to diabetes medication and treatment regimens is a well-recognized challenge for clinicians and other healthcare providers. Often, patients are given prescriptions that they do not fill; they show up unprepared at clinic appointments and they are given instructions that they do not follow or do not comprehend.

To understand why this happens, we need to look at the healthcare system, which strives to be a well-controlled environment, full of highly qualified people speaking very precise and well-understood language and reasoning dispassionately about quantifiable issues with long-term consequences. This environment, however, is the opposite of real life for nearly everybody.

Patients live in an entirely different world: a chaotic environment full of naive reasoning, imprecise or incorrect language, and they are primarily concerned with the qualitative and the trivial minutiae of day-to-day living. Unwittingly, the healthcare system and programs we provide create a narrative where the disease, and not the patient, is the protagonist in the story. Constructing a mechanism whereby the rational and quantifiable objectives of healthcare can be integrated into the emotional and qualitative aspirations of patients is a tremendous challenge, but we do have some new and powerful tools at our disposal that can engage patients in their diabetes management use of gamification.

#### Games versus gamification

Games engage us by providing psychological cues and a meaningful context that is safe but interesting and worth paying attention to. Most of the time our brain is working to figure out what can be ignored in our environment. Therefore, the more time and opportunities you give patients to ignore what you are saying, the more they will take it. To stimulate engagement, patients need to perceive their activities (physical and cognitive) to be interesting, challenging, and worthwhile. Games provide these stimuli.

Gamification works by employing these stimuli outside of a game to gain the user's attention and trust, and to provide feedback that demonstrates that their attention and trust was well placed. However, gamification does not work by adding points, badges, leaderboards, or incentives to make your application look more like a game. This trick will not fool users for long.

Gamification utilizes five key design principles to build engagement within the environment provided. First, patients must be required to make meaningful choices, not fake choices. Second, patients must feel challenged. Challenges must be meaningful and progressively more difficult to sustain engagement. Third, there must be an element of uncertainty; outcomes cannot be predictable. The fourth principle is discovery. Users must learn and master the rules of the game during the course of play. Fifth, the outcomes must be meaningful and perceived as valuable to the user. Although the use of these principles is critical to the mechanics of app development, it is not enough; we incorporate “meaning” into the activity.

#### Importance of creating “meaning”

There are four categories of meaning: social, narrative, aesthetics, and personal progression. Social meaning refers to whether the user perceives the activity as socially appropriate. If other people are engaged in this activity/behavior, then it must be worthwhile. Narrative meaning refers to how users connect with the story: “What is the story, and how did I get here?” We also derive a lot of meaning from aesthetic cues: “What do I see right now that impacts my understanding?” These cues happen below the threshold of consciousness, and it is very important for us to understand what they may be if we are going to deliver tools, systems, and environments that engage people. Meaning also comes from a sense of our personal achievement, how we are progressing.

#### Summary

The “Internet of Things” and new consumer biometric devices paired with smartphones give us new and powerful means of gathering useful data and metadata about patients and their engagement with their care plan. But this is not all these devices can do for us. There is another layer of valuable data that we can gather related to patients' aspirations, intentions, and sentiment. If well used and kept current through regular interactions, these data can help us develop programs and applications that are better able to speak to the social, emotional, and spiritual needs of patients, moment by moment. We have become very good in constructing the “machines” for patients to use to improve their health. Now we must help them find the meaning in the machine.

## Session J: The Global Diabetes Challenges

### Diabetes Management Challenges in Australia

Greg Johnson, Diabetes Australia, Canberra, Australia

#### Background

Diabetes Australia is the national body that represents people with diabetes, families, caregivers, and those at risk in Australia. The national organization was formed in 1957, but local lay organizations date back to 1937. Diabetes Australia works in partnership with diabetes healthcare professionals and educators, researchers, and healthcare providers to minimize the impact of diabetes on the Australian community.

#### Diabetes in Australia

Australia is a large country with a relatively small population of ∼24 million. Most Australians live in the few big cities; however, there are many very isolated and remote communities with small numbers of people throughout the country. The indigenous population is ∼3% of the population. Australia is very multicultural, with approximately one in four born in another country.

More than 1.25 million individuals with diagnosed diabetes are registered on the Australian National Diabetes Services Scheme (NDSS) administered by Diabetes Australia; ∼280 new cases are diagnosed each day. Among those with diagnosed diabetes, 86% have T2D, 10% have T1D, and 3% have gestational or other diabetes. The number of people with undiagnosed diabetes is ∼500,000, and more than 2 million people have prediabetes and are at high risk for developing T2D. Indigenous people in Australia are three to five times more likely to develop diabetes and three to five times more likely to develop the major complications.

In 2010, the cost of diabetes was estimated to be nearly $15 billion Australian dollars. However, costs are expected to double to ∼30 billion Australian dollars by 2025.

#### Progress made

Australia is fiscally challenged like many other countries; however, we have made some progress in recent years. A government-funded CGM access program was established in April 2017. The program provides free access to CGM technology to all children and adolescents up to the age of 21. In 2016, we obtained funding for the NDSS to provide self-management information, education, and support over the next 4 years. In addition, in 2016, a new government-funded program commenced to provide retinal photography screening for people with diabetes, focusing specifically on the indigenous people in rural communities. A number of new medicines have recently been subsidized under the government medicine scheme; a pharmacy-based diabetes screening project is underway, looking at using HbA1c testing in pharmacies for earlier diagnosis of T2D. We are also actively collaborating with national and international organizations to conduct research in diabetes-related psychosocial issues; one in five people with diabetes in Australia experience severe diabetes distress.

Several studies have been conducted to better understand the obstacles to diabetes self-management. Diabetes Australia develops many resource materials (e.g., tool kits) that can be used by primary care practitioners, diabetes educators, and other healthcare professionals in various settings to improve clinician-patient interactions.

### Glycemic Control and Self-Monitoring of Blood Glucose in Chinese Patients with Type 2 Diabetes on Insulin: Results from the COMPASS Study

Linong Ji, Peking University People's Hospital, Beijing, China

#### Background

The clinical role of SMBG in T1D and insulin-treated T2D is well recognized and endorsed in most international and national guidelines of diabetes management. However, the value of SMBG in noninsulin-treated T2D is still being debated. In China, there are an estimated 970,000 individuals with insulin-treated T2D, which makes up ∼30% of the total treated population. Therefore, the potential patient pool of insulin-treated T2D is significant; it is ∼3 million in the next few years. However, the testing frequency of SMBG remains suboptimal in insulin-treated T2D; it is ∼2.5 times/month in the “total treated patients” pool.

#### COMPASS study

##### Design

The COMPASS trial was a 6-month, two-phase, prospective, single-arm, multi-center interventional study. In Phase 1, we assessed the possession rate of blood glucose meters, frequency of SMBG, and glycemic profiles among 3006 patients with insulin-treated T2D. In Phase 2, we assessed the effectiveness of education and behavior intervention in improving SMBG usage and the impact of this intervention on glycemic control among a subset of Phase 1 patients (*n* = 820) with poor glucose control (> 8.0% HbA1c).

##### Phase 1 results

More than 50% of patients with a diabetes duration < 3 years had HbA1c levels of > 8.0%. The average HbA1c levels of patients with a diabetes duration of < 3, 4–6, 7–9, and >10 years were 8.5%, 8.6%, 8.4%, and 8.5%, respectively. The most frequent insulin regimen was with premixed insulin (53.4%), followed by basal insulin only (26.7%), basal plus prandial (13.4%), and prandial insulin only (10.0%). Most patients were also treated with a combination of insulin and oral medication (71.9%). One third of the patients reported that they do not have a blood glucose meter. Among those patients, only 40.9% reported that they always (15.6%) or frequently (25.3%) “follow their blood glucose test results”; 65.8% said that they test “randomly”; and 24.8% of the patients “did not know their blood glucose target.” Patients reported several factors that influenced their infrequent testing. These included: strip cost (37.9%), testing was complicated (28.3%), time constraints (24.4%), and testing was not useful (15.9%). Importantly, 6.6% of patients reported that they did not know how to adjust their insulin based on their test results, often because their physician does not allow them to make insulin adjustments. Approximately 60% of patients reported experiencing hypoglycemia, but that this was a rare occurrence. However, the hypoglycemia rate in patients with >6 months insulin injection (63.3%) was found to be significantly higher than in patients with only 3–6 months of insulin therapy (40.5%), *p* < 0.0001.

##### Phase 2 results

Clinic visits occurred at baseline, months 3 and 6. At baseline, all patients received a free blood glucose meter and test strips; test strip allocation was based on each subject's insulin therapy regimen. Patients were advised to follow a structured SMBG regimen specific to their insulin regimen, and they were trained to respond to SMBG readings via lifestyle changes and insulin dose self-adjustments. Mean (SD) HbA1c levels decreased substantially from baseline at month 3 (Δ −1.81 ± 1.88, *p* < 0.0001) and month 6 (Δ −1.73 ± 1.91, *p* < 0.0001). The percentage of patients who achieved the HbA1c target (< 7.0) was 36% and 40% at months 3 and 6, respectively. Subjects treated with premixed insulin showed the largest decrease in HbA1c from baseline (Δ −1.83% ± 1.85%), followed by those who received an insulin therapy change (Δ −1.67% ± 1.99%), basal plus prandial insulin therapy (Δ −1.62% ± 1.58%), and basal insulin only therapy (Δ −1.51% ± 2.20%). Decreases in mean HbA1c levels were seen in all quartiles according to the duration of T2D and insulin therapy; however, subjects with the shortest duration of diabetes and/or insulin therapy experienced the largest decreases in mean HbA1c. The percentage of patients who performed SMBG at least once daily more than doubled at months 3 and 6. The percentage of patients who experienced hypoglycemia events was lower in the patients who self-adjusted the therapy according to the results of SMBG compared with those who did not (36.5% vs. 41.5%).

#### Summary

There is low utilization of SMBG in insulin-treated T2D Chinese adults, with approximately two-thirds of patients reporting irregular use of SMBG. This correlates with an overall poor level of glycemic control. A structured SMBG regimen, with training on the interpretation of and responses to SMBG readings, has increased SMBG frequency, improved the HbA1c levels and the management of insulin-treated T2D in the study participants. The structured SMBG program used in the study significantly improved QoL outcomes. Physical and emotional role functioning are the two QoL scales that demonstrate the largest improvement with SMBG in this study.

## Session K: Keynote Lecture

### The Promise of Digitalization and Big Data in Healthcare

Marcus Vetter, Mannheim University of Applied Sciences, Mannheim, Germany

#### Background

Digitalization offers enormous potential to processing large amounts of medical data, which could not possibly be examined by an individual healthcare professional in his/her lifetime. Further, it can take this information into consideration in the process of decision making for subsequent diagnoses and treatments. The promise of digitalization or Big Data in healthcare is ultimately based on the automatic evaluation of large amounts of data. For this purpose, “Machine Learning” is the key technology used to extract the knowledge contained in Big Data. “Deep Learning” allows us to utilize this knowledge and is considered the primary breakthrough in the field of artificial intelligence.

#### Deep Learning

Deep Learning architecture utilizes multiple neural networks, which are layered on top of each other. Sophisticated algorithms and powerful graphics processing unit technology utilize these networks in ways that allow computers to both “learn” and handle multiple tasks simultaneously.

Development of Deep Learning can be traced back to Huber and Wilson, who investigated optical cortex functionality in cats in 1959. Findings from this research formed the basis for their theory of hierarchical processing of information in the visual cortex. In 1970, David Marr, a psychologist, computer scientist, and mathematician, developed a model of how human vision works.

Over the past few years, advances in Deep Learning technology have resulted in several product and software innovations, such as driverless cars (Tesla Autopilot 2.0), advanced image and speech recognition capabilities, and others. The architecture of neural networks has also developed rapidly; Microsoft currently has a network with more than 1000 layers.

#### Potential applications in medicine

Although the integration of Deep Learning technology into medicine is relatively new, we are starting to see some potentially valuable applications. For example, Google Deep Mind has partnered with the National Health Service in the United Kingdom for the prevention of blindness, using optical coherence tomography to automatically diagnose diabetic retinopathy and age-related macular degeneration. It is anticipated that Deep Learning will soon enable clinicians to identify relevant disease states so that the patient can be transferred to the treating physician in a timely manner. Deep Learning technology is also able to recognize the relevant health status of patients from the large number of data.

Other applications are also emerging. For example, classification of food and estimation of calorie intake is now possible by using a smartphone. In addition, scientists at Florida University have developed software that can analyze health records for patterns that identify patients who may be at risk for suicide. Facebook claims that it can do similar pattern recognition by analyzing the content of the user's Facebook page. The company has also announced plans to develop software that allows users to enter text into a smartphone directly from the mind. Developers are also exploring Deep Learning technology as a diagnostic tool that can detect unrecognized depression using MRI scans.

Deep Learning is also being applied to prioritizing text information, which would allow clinicians to quickly mark medical records according to relevant criteria, medications, contraindications, etc. Researchers are also exploring the use of smartphones and Deep Learning software to detect vocal patterns that might signal post-traumatic stress syndrome, heart disease and, even, patient adherence to medication regimens.

#### Conclusions

Deep Learning technologies are emerging rapidly and have the potential to improve both the quality and efficiency of healthcare delivery. However, additional research will be needed over the next 10 years. Clinical studies with clear learning objectives for Deep Learning technologies are required, and clear, relevant questions must be formulated by medical experts. Multicenter data collection must be planned and implemented, using the maximum amount of data and advanced statistical methods. It is also important that several experts have access to these data and the ability to assess them independently. Medical disciplines are tasked to conceive the necessary scientific structures and research programs.
